# *Lactococcus lactis* FNBPA^+^ (pValac:*e6ag85a*) Induces Cellular and Humoral Immune Responses After Oral Immunization of Mice

**DOI:** 10.3389/fmicb.2021.676172

**Published:** 2021-05-20

**Authors:** Camila Prósperi de Castro, Bianca Mendes Souza, Pamela Mancha-Agresti, Vanessa Bastos Pereira, Meritxell Zurita-Turk, Tatiane Melo Preisser, Vanessa Pecini da Cunha, Janete Soares Coelho dos Santos, Sophie Yvette Leclercq, Vasco Azevedo, Anderson Miyoshi

**Affiliations:** ^1^Laboratory of Genetic Technology, Department of Genetics, Ecology and Evolution, Institute of Biological Sciences, Federal University of Minas Gerais (UFMG), Belo Horizonte, Brazil; ^2^Laboratory of Cellular and Molecular Genetics, Department of Genetics, Ecology and Evolution, Institute of Biological Sciences, Federal University of Minas Gerais (UFMG), Belo Horizonte, Brazil; ^3^Laboratory of Biotechnological Innovation, Research and Development Directorate, Ezequiel Dias Foundation (FUNED), Belo Horizonte, Brazil

**Keywords:** lactic acid bacteria, *Lactococcus lactis*, DNA vaccine, tuberculosis, ESAT6/Ag85A

## Abstract

The development of a new vaccine strategy against tuberculosis is urgently needed and has been greatly encouraged by the scientific community worldwide. In this work, we constructed a lactococcal DNA vaccine based on the fusion of two *Mycobacterium tuberculosis* antigens, ESAT-6 and Ag85A, and examined its immunogenicity. The coding sequences of the *ESAT-6* and *Ag85A* genes were fused and cloned into the eukaryotic expression pValac vector, and the functionality of the vector was confirmed *in vitro*. Then, *L. lactis* FnBPA^+^ (pValac:*e6ag85a*) was obtained and used for oral immunization of mice. This strain induced significant increases in IFN-γ, TNF-α, and IL-17 cytokines in stimulated splenocyte cultures, and significant production of antigen-specific sIgA was observed in the colonic tissues of immunized mice. We demonstrated that *L. lactis* FnBPA^+^ (pValac:*e6ag85a*) generated a cellular and humoral immune response after oral immunization of mice. The strategy developed in this work may represent an interesting DNA mucosal vaccine candidate against tuberculosis, using the fusion of two highly immunogenic antigens delivered by safe lactic acid bacteria.

## Introduction

Tuberculosis (TB), an infectious disease caused by *Mycobacterium tuberculosis*, remains a serious public health problem worldwide, especially in developing countries. The only vaccine available for clinical use is Bacillus Calmette-Guérin (BCG) and, although BCG is widely used, its efficacy against pulmonary TB is controversial (Kaufmann et al., 2015). Hence, the investigation of strategies for the development of more effective and economically viable vaccines against tuberculosis is necessary and has been greatly encouraged. Over the last few years, the international scientific community has made a substantial effort to search for more effective vaccine alternatives to BCG or to increase its immunogenicity. These alternatives include vaccines based on genetically modified mycobacteria lineages, subunit vaccines and DNA vaccines (Kaufmann et al., 2014). DNA vaccines have the advantages of T cell activation and antibody production without the possible limitations of immunization with live pathogenic microorganisms (Kutzler and Weiner, 2008).

The proteins secreted by *M. tuberculosis* are considered attractive vaccine candidate antigens (Andersen et al., 1991). Among these proteins, two are prominent: ESAT-6 and Ag85A. 6-kDa Early Secreted Antigenic Target (ESAT-6) is an immunodominant antigen secreted in the early stages of infection and is present in pathogenic *M. tuberculosis* but absent in BCG (Pym et al., 2002). Ag85A belongs to the antigen 85 complex (Ag85) and is considered a highly immunogenic virulence factor (Dietrich et al., 2006). Studies have revealed that DNA vaccines using these proteins can increase the T helper cell type 1 (Th1) response, which is critical in protection against tuberculosis (Romano et al., 2006; Xu et al., 2008).

Currently, despite the various available DNA vaccine delivery methods, bacterial mucosal immunization has emerged as a good strategy (Schoen et al., 2004). However, many of these microorganisms are attenuated enteropathogenic bacteria, and the potential risk of reversion may difficult their use (Dunham, 2002). Fortunately, this risk can be circumvented by the use of non-pathogenic bacteria, such as *Lactococcus lactis*.

*Lactococcus lactis*, considered the model lactic acid bacteria, is classified as Generally Recognized As Safe (GRAS) and has been used for production and delivery of antigens and cytokines to mucosal surfaces for a long time (Wells, 2011). The potential of this microorganism as a vehicle for the delivery of DNA vaccines has been investigated and good results were observed (Del Carmen et al., 2013; Pereira et al., 2015; Souza et al., 2016; Mancha-Agresti et al., 2017; Zurita-Turk et al., 2020). These results were achieved by the use of the pValac vector (Guimarães et al., 2009) and the recombinant strain *L. lactis* FnBPA^+^ that expresses fibronectin binding protein A (FnBPA) of *Staphylococcus aureus*, an invasin involved in adhesion and invasion of eukaryotic cells (Que et al., 2001). *In vitro* and *in vivo* assays demonstrated the invasiveness of *L. lactis* FnBPA^+^ and its ability to deliver the pValac vector to eukaryotic cells (Innocentin et al., 2009; Pontes et al., 2012; Almeida et al., 2014).

Thus, our aim was to construct *L. lactis* FnBPA^+^ harboring the pValac vector containing the fusion ORF *ESAT6*-*Ag85A* of *M. tuberculosis* and use this system for oral immunization of mice. Moreover, we aimed to evaluate the immune response generated by this vaccine strategy.

## Materials and Methods

### Bacterial Strains, Plasmids, and Growth Conditions

The plasmid and bacterial strains used are listed in Table 1. The pValac plasmid was constructed by our group in 2009 (Guimarães et al., 2009). The *L. lactis* FnBPA^+^ lineage was developed by Que et al. (2001), who kindly gave it to the group of Dr. Philippe Langella from INRAe—France. Dr. Philippe Langella passed it on to us and, currently, both the plasmid and the lineage are part of our microbiological collection.

**Table 1:** Bacterial strains and plasmids.

**Bacterial**	**Characteristics**	**References**
*Escherichia coli* TG1	*E. coli* K-12-derived strain	Lucigen
*Escherichia coli* TG1 (pValac:*gfp*)	*E. coli* TG1 carrying the pValac:*gfp*^*a*^ plasmid	Guimarães et al., 2009
*Escherichia coli* TG1 (pValac:*e6ag85a*)	*E. coli* TG1 carrying the pValac:*e6ag85a* plasmid	This work
*Lactococcus lactis* FnBPA^+^	*L. lactis* MG1363 strain expressing FnBPA^*b*^ of *Staphylococcus aureus*	Que et al., 2001
*Lactococcus lactis* FnBPA^+^ (pValac:*e6ag85a*)	*L. lactis* MG1363 strain expressing FnBPA of *S. aureus* carrying the pValac:*e6ag85a* plasmid	This work
**Plasmid**	**Characteristics**	**References**
pValac:*gfp*	Eukaryotic expression vector (pCMV^c^ /Cm(r)^d^ /RepA and RepC^e^) containing the coding sequence for GFP	Guimarães et al., 2009
pOri23-*fnbA*	*L. lactis-E. coli* shuttle vector containing the coding sequence for FnBPA (Ery^r^)^f^	Que et al., 2001
pValac:*e6ag85a*	pValac vector containing the coding sequence for fusion protein ESAT-6/Ag85A of *Mycobacterium tuberculosis*	This work

a *gfp, green fluorescent protein;*

b *FnBPA, fibronectin binding protein;*

c *pCMV, cytomegalovirus promoter;*

d *Cm^r^, chloramphenicol resistance gene;*

e *RepA and RepC, replication origins;*

f *Ery^r^, erythromycin resistance gene*.

*Escherichia coli* TG1 was grown in LB broth (Acumedia, San Bernardino, United States) at 37°C with shaking. *Lactococcus lactis* FnBPA^+^ was grown at 30°C without shaking in M17 broth (Sigma-Aldrich, Darmstadt, Germany) enriched with 0.5% glucose (GM17). Recombinant bacteria were selected by addition of the following antibiotics: for *L. lactis* FnBPA^+^, erythromycin at 5 μg/ml; for *L. lactis* FnBPA^+^ (pValac*:e6ag85a*), erythromycin at 5 μg/ml and chloramphenicol at 10 μg/ml; and for *E. coli* (pValac*:e6ag85a*), chloramphenicol at 10 μg/ml.

Plasmids from *E. coli* and *L. lactis* were isolated as described by Green and Sambrook (2012), with the following modification: for *L. lactis*, TE-LYS (25% sucrose, 1 mmol EDTA, 50 mmol Tris–HCl, pH 8, lysozyme 10 mg/ml) was added to the samples for 1 h at 37°C before the addition of lysis solution.

### DNA Vaccine Construction

The *ESAT-6* and the *Ag85A* coding sequences were amplified by PCR using Pfx Platinum^®^ High-Fidelity DNA Polymerase (Life Technologies, Carlsbad, United States) and the genomic DNA of *M. tuberculosis* H37Rv strain (ATCC 27294) as a template. The oligonucleotides used for *ESAT-6* were as follows: 5′ - CGGGATCC**CCACCATG**GAGCAGCAGTGGAATTTCGCG-3′ (forward) containing the *Bam*HI restriction site (underlined) and the customized Kozak sequence (in bold) and 5′ - CTAGTCTAGATGCGAACATCCCAGTGACG-3′ (reverse) containing the *Xba*I restriction site (underlined). The reverse primer was designed without a stop codon. The oligonucleotides used for *Ag85A* were as follows: 5′ -CTAGTCTAGAATGCAGCTTGTTGACAGGGTTC-3′ (forward) containing the *Xba*I restriction site (underlined) and 5′ CGGAATTCCTAGGCGCCCTGGGGCGC-3′ (reverse) containing the *Eco*RI restriction site (underlined).

PCR products were purified from agarose gels using illustra™ GFX™ PCR DNA and Gel Band Purification kit (GE Healthcare, Chicago, United States) and individually digested with *Xba*I. Then, fragments were ligated using T4 DNA ligase (Life Technologies, Carlsbad, United States). The fusion fragment *ESAT6-Ag85A* was digested with *Bam*HI and *Eco*RI enzymes and inserted into the corresponding sites of the eukaryotic expression vector pValac (Guimarães et al., 2009), which was previously cleaved with the same restriction enzymes.

**Figure 1:**
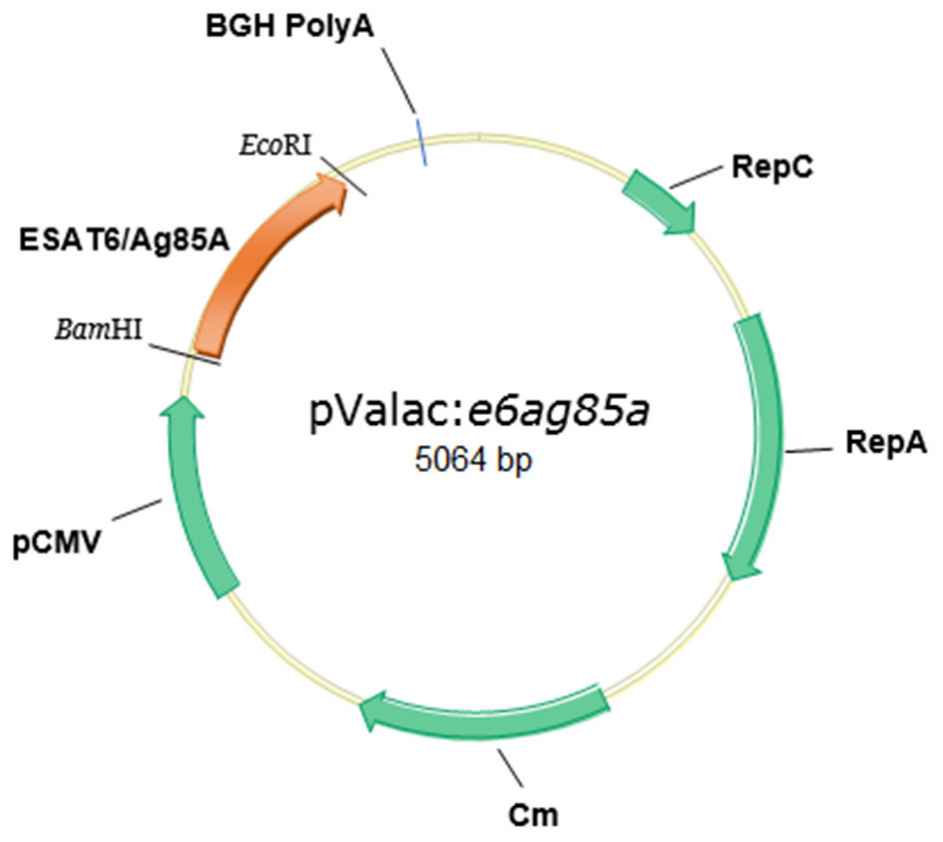
Structure of the pValac:*e6ag85a* vector. Tick marks indicate *Bam*HI and *Eco*RI restriction sites and BGH polyadenylation region (polyA). Arrows indicate cytomegalovirus promoter (pCMV), ORF of the ESAT6/Ag85A fusion protein, replication origin of *E. coli* (RepC), *L. lactis* (RepA), and chloramphenicol resistance gene (Cm).

The pValac:*e6ag85a* plasmid (Figure 1) was transformed into *E. coli* TG1, producing the *E. coli* TG1 (pValac:*e6ag85a)* strain. The insert integrity was confirmed by DNA sequence analysis, using a BigDye Terminator v3.1 Cycle Sequencing kit (Applied Biosystems, Foster City, United States) and ABI3130 sequencing equipment (Applied Biosystems, Foster City, United States). Finally, pValac:*e6ag85a* was transformed by electroporation (Langella et al., 1993) into *L. lactis* FnBPA^+^, producing the *L. lactis* FnBPA^+^ (pValac:*e6ag85a*) strain.

### Cell Transfection and Protein Expression Assays

The pValac:*e6ag85a* vector was transfected into the Chinese hamster ovarian cell line (Flp-In™-CHO; Life Technologies, Carlsbad, United States) to evaluate its functionality by analyzing the expression of the protein by confocal microscopy and flow cytometry. CHO cells were cultured in F12 Ham media (Gibco, Dublin, Ireland) supplemented with 10% fetal bovine serum, 1% L-glutamine, zeocin (100 ng/ml) and 2.5% HEPES [4-(2-hydroxyethyl)-1-piperazineethanesulfonic acid]. The cells were transfected with 4 μg of pValac:*e6ag85a* vector using Lipofectamine™ 2000 transfection reagent (Life Technologies, Carlsbad, United States) as described by the supplier. Cells that received the pValac:*gfp* vector served as the positive control; cells that did not receive plasmids served as the negative control. DNA plasmids used in transfection were prepared using Qiagen^®^ Plasmid Midi kit (Qiagen, Hilden, Germany), according to the manufacturer's instruction.

Forty-eight hours post transfection, pValac:*e6ag85a*-transfected and control cells were fixed with 4% paraformaldehyde (Sigma-Aldrich, Darmstadt, Germany) for 15 min and permeabilized with 0.1% Triton X-100 (Sigma-Aldrich, Darmstadt, Germany) for 10 min. Then, the cells were incubated for 2 h with a specific mouse monoclonal immunoglobulin G1 (IgG1) anti-ESAT-6 antibody 1 mg/ml, diluted 1/1000 in PBS/BSA 1% (Abcam ab26246; Abcam, Cambridge, United Kingdom). Next, the cells were incubated with goat anti-mouse IgG (H+L) Alexa Fluor^®^ 488 (4 μg/ml, diluted 1/500 in PBS/BSA 1%; Life Technologies, Carlsbad, United States) for 1 h in darkness. Nuclear labeling was performed concurrently with secondary antibody labeling by incubation with the fluorochrome DAPI 2 μg/ml (Life Technologies, Carlsbad, United States), diluted 1:300 in PBS/BSA 1%. Samples were mounted and images were captured using a Zeiss LSM 510 META inverted confocal laser-scanning microscope (Zeiss, Oberkochen, Germany). Images were collected and analyzed using Zeiss LSM Image Browser software (Zeiss, Oberkochen, Germany).

For flow cytometry analysis, 1 × 10^6^ pValac:*e6ag85a*-transfected and control cells (non-transfected cells) were fixed and permeabilized using a Mouse Foxp3 Buffer Set kit (BD Pharmingen^TM^, San Jose, United States). The cells were then incubated for 30 min with mouse monoclonal IgG1 anti-ESAT-6 antibody Abcam ab26246 diluted 1/1000 in PBS/BSA 1% or with mouse monoclonal anti-Ag85A antibody (H-2b haplotype mice—Mab DT-17/4, source: Professor Kris Huygens, Pasteur Institute of Brussels, Belgium) diluted 1/50 in PBS/BSA 1%. Next, the cells were incubated with secondary goat anti-mouse IgG (H+L) Alexa Fluor^®^ 488 diluted 1/500 in PBS/BSA 1% for 30 min. Finally, the cells were centrifuged and suspended in PBS, and quantification of ESAT-6- and Ag85A-producing CHO cells was performed by BD Accuri™ C6 Flow Cytometer equipment (BD Biosciences, San Jose, United States). The acquired data were analyzed using FCAP array software (BD Biosciences, San Jose, United States).

### Animals and Ethical Approval

Conventional female BALB/c mice (6–7 weeks old) were used for the immunization assays. Mice were obtained from the Central Bioterium of Federal University of Minas Gerais (UFMG, Belo Horizonte, Brazil). They were maintained in mini-isolators housed in ventilated racks with controlled conditions (temperature of 22 ± 2°C, humidity 50 ± 10%, air flow 35 exchanges/hour and light exposure 12-h light/dark cycle) and free access to water and rodent food. All the experiments and handling of the mice were performed in accordance with the ethical principles for animal experimentation adopted by the Animal Use Ethics Commission (CEUA, Registration Number 66/11) of Federal University of Minas Gerais (UFMG, Belo Horizonte, Brazil).

### Immunization

BALB/c mice were randomly divided into the following experimental groups: negative control (saline 0.9%) (-), *L. lactis* FnBPA^+^ (invasive *L. lactis* strain, negative control) (FN) and *L. lactis* FnBPA^+^ (pValac:*e6ag85a*) (FEA). Each animal was orally immunized by gavage with 1 × 10^8^ CFU in a final volume of 100 μl of saline 0.9%. The immunizations were performed at three different time points (days 1, 15, and 29) for three consecutive days at each time point. On day 43, all animals were anesthetized by intraperitoneal injection with a ketamine (60 mg/kg) and xylazine (8 mg/kg) cocktail and then euthanized. Two individual experiments were performed for each individual protocol (six animals/group).

### Cytokine Measurement

Cytokine levels in the splenocyte supernatants were measured using a commercially available enzyme-linked immunosorbent assay (ELISA) kit (R&D DuoSet kit; R&D Systems^TM^, Minneapolis, United States) according to the manufacturer's instructions to characterize the cellular immune response profile. Briefly, spleens were aseptically removed on the day of sacrifice and macerated; red blood cells were lysed. A total of 1 × 10^6^ splenocytes from each immunized animal were plated (96-well microtiter plates) in complete RPMI-1640 (Sigma-Aldrich, Darmstadt, Germany) medium [10% fetal bovine serum (Gibco, Dublin, Ireland), sodium pyruvate 1 mmol, non-essential amino acids 1 mmol (Gibco, Dublin, Ireland), gentamicin 25 μg/ml (Gibco, Dublin, Ireland), and L-glutamine 2 mmol (LGC Biotecnologia, Cotia, Brazil)]. These cells were submitted to treatment with only RPMI (non-stimulated cells; negative control) or with RPMI containing 5 μg/ml of rESAT-6 (Pereira et al., 2015) and then incubated at 37°C in 5% CO_2_. As a positive control, one sample of each experimental group was stimulated with RPMI containing Concanavalin A (ConA—Sigma-Aldrich, Darmstadt, Germany) 16 μg/ml (data not shown).

Cell supernatants were collected to measure the levels of the cytokines interferon-gamma (INF-γ), tumor necrosis factor alpha (TNF-α), interleukin 17 (IL-17), 10 (IL-10), and 4 (IL-4) by sandwich ELISA using the described kit, after 60 hours of stimulation. Absorbance was measured at 492 nm using an Expert Plus Microplate Reader (Biochrom Asys, Cambridge, United Kingdom). Results for all cytokines were calculated by subtracting the basal values of cytokines from cells that received only RPMI medium from the values of cytokines from rESAT-6-stimulated cells.

### Characterization of the Humoral Immune Response Pattern

The humoral immune response of immunized animals was evaluated by measuring mucosal anti-ESAT-6 sIgA levels in macerated mouse colons. For this, colons were macerated in buffer solution containing anti-proteases in a ratio of 1 ml of solution to 100 mg of tissue. The IKA T10 Basic Homogenizer workcenter (IKA^®^ Brasil, Campinas, Brazil) was employed for this purpose. The homogenates were centrifuged, the supernatant was collected and the measurement of sIgA was performed by indirect ELISA as follows. Recombinant ESAT-6 (5 μg/ml) was used to coat microliter plates overnight (Pereira et al., 2015). Then, samples without dilution were added and incubated for 1 h. Next, horseradish peroxidase (HRP)-conjugated goat anti-mouse antibodies (Sigma-Aldrich, Darmstadt, Germany) diluted in PBS/casein (1/8000) were added and incubated for 2 h. Finally, orthophenylenediamine (OPD−1 mg/ml; Sigma Aldrich, Darmstadt, Germany) was used for color development as an indicator. Absorbance was measured at 492 nm using an ELISA Expert Plus Microplate Reader (Biochrom Asys, Cambridge, United Kingdom).

### Statistical Analysis

Statistical analyses were performed using one-way analysis of variance (ANOVA) followed by Bonferroni post-test using GraphPad Prism 5.0 software (San Diego, CA, United States). The data are expressed as the mean ± standard error of the mean (SEM); *p* < 0.05, *p* < 0.01, and *p* < 0.001 were considered statistically significant.

## Results

### Construction of the Recombinant Strain *L. lactis* FnBPA^+^ (pValac:*e6ag85a)*

The fused ORF *ESAT6-Ag85A* (1,322 bp) (Gen Bank number *ESAT-6*: 886209; *Ag85A*: 886132) was directionally cloned into the pValac vector. pValac comprises the cytomegalovirus promoter (pCMV), the polyadenylation sequence of bovine growth hormone (BGH) (unit of eukaryotic expression), the origins of replication for both *E. coli* and *L. lactis*, and the chloramphenicol (Cm) resistance gene (prokaryotic region). The final pValac:*e6ag85a* construct (Figure 1) was confirmed by molecular biology methods such as PCR, enzymatic digestion, and sequencing (data not shown). Recombinant *L. lactis* FnBPA^+^ (pValac:*e6ag85a*) was constructed by transformation of the invasive *L. lactis* FnBPA^+^ strain with the pValac:*e6ag85a* plasmid.

### Eukaryotic Cells Transfected With the Plasmid pValac:*e6ag85a* Can Express ESAT6-Ag85A Protein

**Figure 2:**
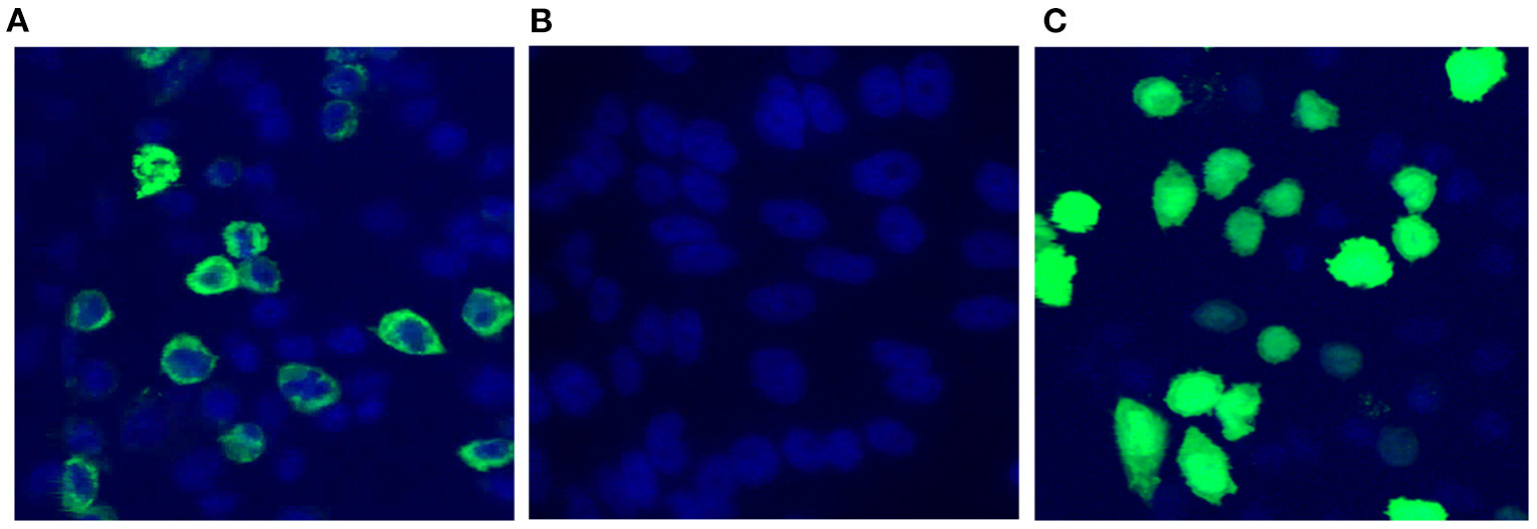
Confirmation of pValac:*e6ag85a* functionality. Confocal Microscopy. **(A)** CHO cells transfected with pValac:*e6ag85a* labeled with anti-ESAT-6 primary antibody, Alexa 488 secondary antibody and DAPI; **(B)** Untransfected CHO cells labeled with anti-ESAT-6 primary antibody, Alexa 488 secondary antibody and DAPI (negative control); **(C)** CHO cells transfected with pValac:*gfp* labeled with DAPI (positive control). Images obtained using a Zeiss LSM 510 META inverted confocal laser-scanning microscope with a 63X objective.

The functionality of pValac:*e6ag85a* was confirmed by confocal microscopy and flow cytometry. In confocal microscopy analysis, pValac:*e6ag85a*-transfected CHO cells labeled with a specific mouse anti-ESAT-6 monoclonal antibody and secondary antibody conjugated to Alexa 488 showed green fluorescence in the cytoplasm, thus confirming the expression of ESAT-6 protein (Figure 2A). Protein expression was not detected in untransfected CHO cells labeled with the same primary and secondary antibodies (negative control) (Figure 2B). Cells transfected with the pValac:*gfp* plasmid were assayed as a positive control, which showed specific green fluorescence protein (GFP) expression (Figure 2C).

**Figure 3:**
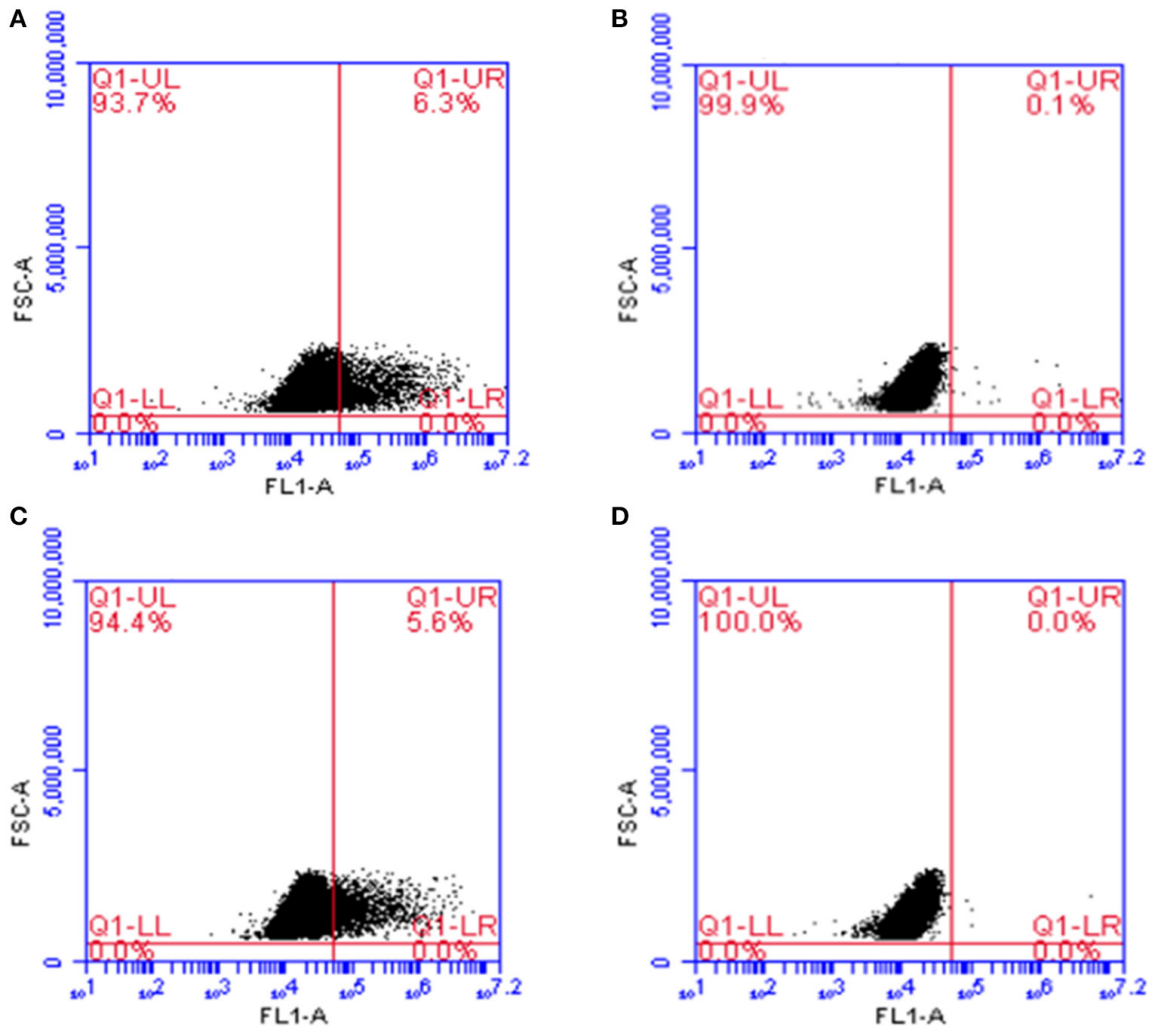
Expression of ESAT-6 and Ag85A proteins by CHO cells transfected with the pValac:*e6ag85a* plasmid. Flow Cytometry. **(A)** Transfected cells labeled with primary anti-ESAT-6 and secondary Alexa 488 antibodies; **(B)** Non-transfected cells labeled with primary anti-ESAT-6 and secondary Alexa 488 antibodies; **(C)** Transfected cells labeled with primary anti-Ag85A and secondary Alexa 488 antibodies; **(D)** Non-transfected cells labeled with primary anti-Ag85A and secondary Alexa 488 antibodies. Images obtained using *FCAP array software* (BD Biosciences).

In flow cytometry, cells transfected with the plasmid pValac:*e6ag85a* and labeled with the antibody against ESAT-6 (Figure 3A) or Ag85A (Figure 3C) antigens showed protein expression in 6.3 and 5.6% of the analyzed events, respectively. Indeed, no expression was observed in non-transfected cells (Figures 3B,D). Taken together, these results confirmed the functionality of the pValac:*e6ag85a* vector.

### Oral Immunization With *L. lactis* FnBPA^+^ (pValac:*e6ag85a*) Can Induce IFN-γ, TNF-α, and IL-17 Cytokines

**Figure 4:**
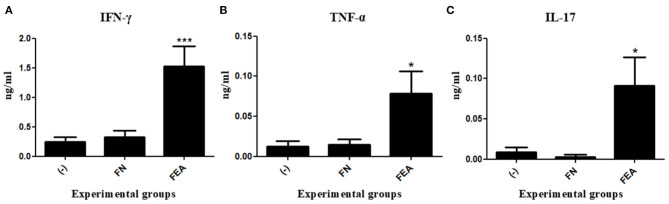
Production of cytokines after oral immunization of mice. Levels of **(A)** IFN-γ, **(B)** TNF-α, and **(C)** IL-17 in the supernatant of splenocyte cultures stimulated with rESAT-6 and analyzed by ELISA. Experimental groups: Saline (-; negative control), *Lactococcus lactis* FnBPA^+^ (FN; negative control), and *Lactococcus lactis* FnBPA^+^ (pValac:*e6ag85a*) (FEA). Data are shown as the mean ± SEM of two independent experiments (*n* = 12). *P < 0.05 or ***P < 0.001.

To determine the cellular immune response pattern after mouse immunization, we measured the levels of IFN-γ, TNF-α, IL-17, IL-4, and IL-10 cytokines in the supernatant of rESAT-6-stimulated splenocytes. Compared with control mice immunized with *L. lactis* FnBPA^+^ (FN) or saline (-), mice immunized with *L. lactis* FnBPA^+^ (pValac:*e6ag85a*) (FEA) exhibited a significant increase in IFN-γ and TNF- α levels (Figures 4A,B).

Regarding IL-17, mice immunized with *L. lactis* FnBPA^+^ (pValac:*e6ag85a*) showed significantly higher levels of this cytokine than did mice immunized with *L. lactis* FnBPA^+^ or saline (Figure 4C). The other cytokines measured, IL-4 and IL- 10, which are specific to the Th2 immune response, were not detected in the supernatants of the splenocytes culture.

### Increased sIgA Is Detected After Immunization With *L. lactis* FnBPA^+^ (pValac:*e6ag85a*)

**Figure 5:**
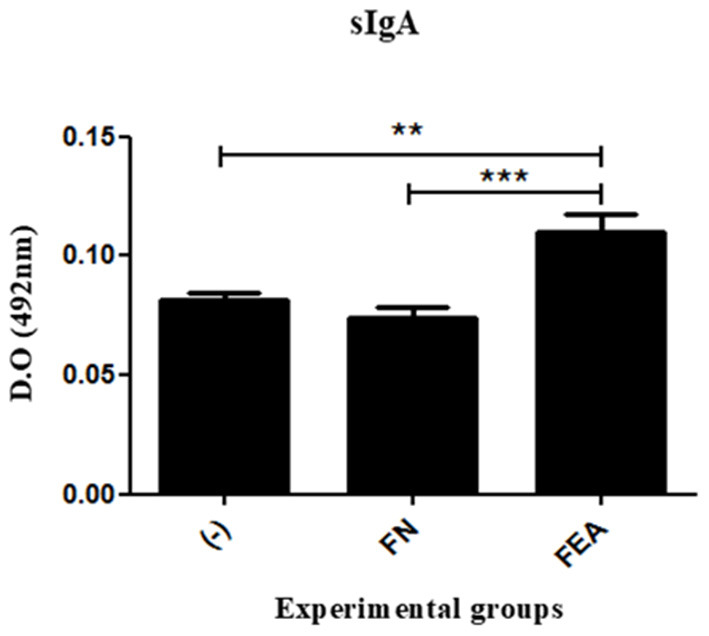
Production of sIgA after oral immunization of mice. Levels of anti-ESAT6 sIgA in the colonic tissues of mice orally immunized with *L. lactis* FnBPA^+^ (pValac:*e6ag85a*) and analyzed by ELISA. Experimental groups: Saline (-; negative control), *Lactococcus lactis* FnBPA^+^ (FN; negative control), and *Lactococcus lactis* FnBPA^+^ (pValac:*e6ag85a*) (FEA). Data are shown as the mean ± SEM of two independent experiments (*n* = 12). **P < 0.01 or ***P < 0.001.

The principal immunoglobulin in mucosa immunity is secretory IgA (sIgA). The results obtained in this study showed that anti-ESAT-6 sIgA levels in the macerated colons of mice immunized with *L. lactis* FnBPA^+^ (pValac:*e6ag85a*) were significantly higher than those observed in the colons of mice immunized with *L. lactis* FnBPA^+^ or saline (Figure 5). Indeed, these results demonstrated that administration of the *L. lactis* FnBPA^+^ (pValac:*e6ag85a*) strain induced a mucosal immune response *in vivo*.

## Discussion

The use of *Lactococcus lactis* FnBPA^+^ for mucosal DNA delivery is a promising strategy for immunization. The main mucosae exploited for immunization with DNA vaccines are intranasal (Dou et al., 2012) and oral (Wang et al., 2009; Pereira et al., 2015, 2017). Perhaps the most advantageous is the oral route, considering that the gastrointestinal tract contains more cells of the immune system than any other tissue, representing the largest immunological compartment of the organism (Mowat and Agace, 2014). Thus, the development of DNA vaccines suitable for oral immunization, aiming at reaching the intestinal mucosa and, consequently, their inherent immunological advantages, is particularly interesting.

Recently, using the same vaccination strategy employed in this work but exploring only the role of ESAT-6 as a candidate antigen, Pereira et al. reported a significant increase in IFN-γ production in mice orally immunized with *L. lactis* FnBPA^+^ (pValac:*esat6*). However, no differences were observed in the expression of other equally important cytokines, such as TNF-α, in the immune response against *M. tuberculosis* (Pereira et al., 2015).

Moreover, Mancha-Agresti et al. (2017) developed the *L. lactis* FnBPA^+^ (pValac:*ag85a*) DNA vaccine, which encodes the Ag85A antigen. Following intranasal administration of the vaccine to mice, they found a significant increase in production of IFN-γ and TNF-α by the animals, similar to the present study, but with smaller magnitudes. In addition, the increase in IL-17, which is important in the early stages of TB control, was not verified (Mancha-Agresti et al., 2017).

In this study, we used the coding sequence of these two highly immunogenic proteins in a construct in which they were fused and cloned into the pValac vector. The pValac:*e6ag85a* functionality was confirmed, and this plasmid was transformed into the invasive *L. lactis* FnBPA^+^ strain. *L. lactis* FnBPA^+^ (pValac:*e6ag85a*) was used to immunize BALB/c mice by the oral route, and cellular and humoral immune responses were evaluated.

Concerning the cellular immune response, compared with the control groups, the group immunized with the *L. lactis* FnBPA^+^ (pValac:*e6ag85a*) strain showed increased IFN-γ, TNF-α, and IL-17 levels. IFN-γ is considered the most relevant cytokine in the immune response against *M. tuberculosis* and is mainly secreted in response to the activation of naive CD4^+^ T cells in Th1-type effector cells. Th1 cells represent a primordial defense against intracellular microorganisms, such as *M. tuberculosis* (Lyadova and Panteleev, 2015). In a previous study, mice deficient in IFN-γ died in the early stages of *M. tuberculosis* infection and presented a high mycobacterial burden in their bodies (Cooper et al., 1993). Similar effects have been observed in humans who have defective IFN-γ signaling pathways, which results in severe infections caused by this microorganism (van de Vosse et al., 2004).

TNF-α, in turn, is a potent activator of macrophages, acting in synergy with IFN-γ in the induction of its antimicrobial activity (Mootoo et al., 2009). Knock-out mice for TNF-α developed a lethal *M. tuberculosis* infection (Jacobs et al., 2000). In humans, the importance of TNF-α has been demonstrated notably by the increased risk of reactivation of latent TB in patients with chronic inflammatory diseases who underwent anti-TNF-α therapies (Feldmann and Maini, 2001; Harris and Keane, 2010).

The IL-17 cytokine, secreted by Th17 cells, acts on the stimulation of the granulocytic lineage, the recruitment of neutrophils to sites of infection and the induction of pro-inflammatory molecules secretion (Lyadova and Panteleev, 2015). In the human bronchial epithelium, Th17 cells induce the expression of antimicrobial mucins and peptides, which together with neutrophil activity, provide mechanisms to combat *M. tuberculosis* (Kao et al., 2004; Tsai et al., 2013). Low levels of IL-17 in TB patient serum is related to high mortality (Lyadova and Panteleev, 2015).

Taken together, these findings support the concept that the generation of Th1 and Th17 cellular immune response profiles with production of their signature cytokines is necessary for TB defense; these patterns were observed in this work. When comparing the results of the present study with those obtained by Mancha-Agresti et al. (2017) and Pereira et al. (2015), we believe that the results obtained here are due to a greater amount of target epitopes coexisting in the same vaccine plasmid, which generated stronger immune responses.

Regarding the mucosal immune response generated after vaccination with *L. lactis* FnBPA^+^ (pValac:*e6ag85a*), there was a significant difference between the group that received the DNA vaccine and the control groups. Among the functions of this immunoglobulin, the neutralization of antigens and pathogens in the extracellular environment, with the consequent inhibition of mucosal colonization (Corthésy, 2013), is notable. Furthermore, IgA knock-out mice were more susceptible to infection, which was evidenced by the high mycobacterial load found in the lungs of these animals (Williams et al., 2004; Rodriguez et al., 2005). Thus, a vaccine that induces antigen-specific sIgA responses is particularly desirable for protective immunity, especially for diseases in which the mucosa is their primary site of induction, as is the case of tuberculosis.

In other studies that adopted the approach of multigenic vaccines, results similar to those of this study were obtained. Wang et al. constructed a DNA vaccine encoding a fusion protein composed of ESAT-6 and Ag85B antigens (belonging to the same Ag85A family), which was orally delivered to mice through an attenuated strain of *Salmonella typhimurium*. The animals immunized with this vaccine showed high production of antigen-specific IFN-γ as well as increased levels of sIgA and were protected from a *M. tuberculosis* challenge (Wang et al., 2009). Although interesting, this strategy used pathogenic bacteria as a DNA delivery vector; despite attenuation of the pathogenicity, the risks of reversion to its wild phenotype were not eliminated, which may restrict its use.

Dou et al. tested a DNA vaccine encoding the Ag85A/ESAT-6 fusion protein in association with the pro-inflammatory cytokine IL-21. They used a prime boost scheme, with naked DNA intranasally administered first, followed by BCG as the booster. The researchers observed that vaccinated animals had elevated levels of IFN-γ, measured in the supernatant of the splenocyte culture, as well as activation of the cytotoxic activity of NK cells and increased levels of sIgA in the bronchoalveolar lavage. This vaccine also demonstrated protection against a challenge with *M. tuberculosis* (Dou et al., 2012). However, naked DNA delivery may expose the plasmids to a nuclease-rich environment, which may eventually degrade them, rendering the process unfeasible.

Our vaccine strategy can, then, overcome the abovementioned obstacles since it uses bacteria with a recognized safety profile as a delivery vector for the DNA vaccine. In this way, the plasmid is protected against degradation by nucleases, which may lower the required dose for administration. In addition, the oral route explored in this work allows the DNA vaccine to induce a mucosal immune response, reaching the intestinal mucosa, which is considered the body's largest immune compartment (Mowat and Agace, 2014). In turn, mucosal immunization is extremely important for defense against pathogens that access the host organism through this route, as is the case of *M. tuberculosis*.

In conclusion, *L. lactis* FnBPA^+^ (pValac:*e6ag85a*) was functional after oral immunization and presented features indicative of its potential protective capacity; these results have encouraged us to perform challenge trials with *M. tuberculosis* in the future.

## Data Availability Statement

The datasets presented in this study can be found in online repositories. The names of the repository/repositories and accession number(s) can be found in the article/supplementary material.

## Ethics Statement

The animal study was reviewed and approved by Animal Use Ethics Commission (CEUA, Registration Number 66/11) of Federal University of Minas Gerais (UFMG, Belo Horizonte, Brazil).

## Author Contributions

CC contributed to experimental work, data analysis, manuscript writing, and editing. BS contributed to experimental work, manuscript writing, and revision. PM-A contributed to experimental work and data analysis. VP, MZ-T, TP, VC, JS, and SL contributed to experimental work. VA contributed to conception and design of the study. AM contributed to conception and design of the study and manuscript revision. All authors contributed to the article and approved the submitted version.

## Conflict of Interest

The authors declare that the research was conducted in the absence of any commercial or financial relationships that could be construed as a potential conflict of interest.
